# Comparison of surgical dose response between divergence insufficiency esotropia and non-accommodative esotropia without divergence insufficiency

**DOI:** 10.1371/journal.pone.0220201

**Published:** 2019-07-24

**Authors:** Kyung-Ah Park, Ga-In Lee, Sei Yeul Oh

**Affiliations:** Department of Ophthalmology, Samsung Medical Center, Sungkyunkwan University School of Medicine, Seoul, Korea; Faculty of Medicine, Cairo University, EGYPT

## Abstract

**Background:**

The study compared the surgical dose-response relationship for divergence insufficiency esotropia (DIE) and non-accommodative esotropia without divergence insufficiency (non-DIE).

**Methods:**

We carried out a retrospective review of a group of patients with DIE and non-DIE who underwent recession surgery of the medial rectus muscle in both eyes. Linear regression analysis compared surgical dose–response between the two groups.

**Results:**

In the 15 patients with DIE, the mean preoperative ocular deviation at distant fixation was 25 prism diopters (PD), compared with 3 PD postoperatively. In the 38 non-DIE patients, the mean preoperative ocular deviation was 28 PD, compared with 3 PD postoperatively. The average surgical dose–response was 1.56 PD/mm in the DIE group and 2.91 PD/mm in the non-DIE group (*p* < 0.001).

**Conclusions:**

Surgical dose–response was significantly lower in DIE patients than in non-DIE patients. Augmented MR recession surgery could be regarded as an effective treatment option for patients with DIE. Further study with a larger sample and long-term follow-up is needed to seek the proper extent of surgery in these patients.

## Introduction

Divergence insufficiency esotropia (DIE), originally described by Duane in 1896, is an acquired condition characterized by an esodeviation that measures at least 10 prism diopters (PD) greater at distant rather than near fixation [1]. Patients with DIE complain about double vision at distance. Treatment options include monocular occlusion, base-out prism glasses [2], and a variety of surgical procedures to the horizontal rectus muscles [1,3–6]. The standard surgical treatment is lateral rectus resections or medial rectus (MR) recessions. Both procedures have similar successful outcomes [3,7,8]. Regarding the amount of recession or resection of the extraocular muscles, studies have reported a lower surgical dose-response to MR recession [8–11]. Ridley-Lane et al. reported that augmented amounts were effective [9].

The purpose of this study was to compare the surgical dose–response relationship of DIE to that of non-accommodative esotropia without divergence insufficiency (non-DIE).

## Methods

We carried out a retrospective review of a group of patients who underwent strabismus surgery for esotropia at Samsung Medical Center between January 2012 and January 2017. The Institutional Review Board of Samsung Medical Center approved the study and waived informed consent. We considered for inclusion in the study patients with a distance esotropia ≥10 prism diopters (PD), normal horizontal saccades, and MR recession surgery on both eyes by two surgeons (S.O. and K.P.). Patients with paralytic or restrictive strabismus, previous extraocular muscle surgery, or other ophthalmic or systemic disease or neurological disorder that could affect ocular alignment were excluded.

We defined DIE as a distance esodeviation at least 10 PD greater at distant than at near fixation, symptomatic diplopia at far distance, and fusion at near fixation. Inclusion criteria for non-DIE was no difference in esodeviation between distant and near fixation or not exceeding 4 PD greater at distant fixation than at near fixation.

At the initial visit, all patients underwent a full ophthalmologic assessment, including visual acuity examination, refraction examination, evaluations of ocular alignment status, slit-lamp biomicroscopy, and fundus examination. Ocular alignment was tested by the cover test, the cover-uncover test, and alternate prism cover test. For fixation, a 6/9 visual acuity symbol was used. Neuroimaging was performed in all DIE patients and non-DIE patients with diplopia. Imaging was not routinely performed in non-DIE patients without diplopia.

Because of the experience of undercorrection in many young or middle-aged patients with DIE, we applied largely augmented amounts of surgery in MR muscle recession in DIE patients (Table 1). We applied relatively mild augmentation in non-DIE patients, as described in Table 1. Surgery was based on the largest angle of deviation measured with the alternate prism cover test during distant fixation at the final preoperative visit, which was within one week of surgery. All patients underwent conventional recession surgery of the medial rectus muscle in both eyes symmetrically using 6–0 polyglactin sutures (Vicryl, Ethicon, Johnson and Johnson, Somerville, NJ, USA). All surgeries were performed by two surgeons (K.P. and S.O.). We carried out adjustment in the unilateral eye in all patients who were cooperative with and willing to get this procedure after a detailed explanation of the technique. We used a bow-tie technique [12] to carry out the adjustment, which was performed in the seated upright position with any required refractive correction, with targets at near and distance about four hours after the first surgery. The amount of adjustment was recorded in the medical chart and we did the statistical analysis using the final amount of surgery after adjustment.

**Table 1:** Amount of bilateral medial rectus muscle recession for divergence insufficiency esotropia and non-accommodative esotropia without divergence insufficiency (mm).

Preoperative deviation	DIE	Non-DIE
	(Total amount of surgery)	(Total amount of surgery)
20 PD	7.0 (14.0)	4.5 (9.0)
25 PD	7.0 (14.0)	5.0 (10.0)
30 PD	7.5 (15.0)	5.5 (11.0)
40 PD	7.5 (15.0)	6.0 (12.0)
50 PD	7.5 (15.0)	6.5 (13.0)
60 PD	8.0 (16.0)	7.0 (14.0)

DIE, divergence insufficiency esotropia; Non-DIE, non-accommodative esotropia without divergence insufficiency; PD, prism diopters

Data on follow-up examinations after strabismus surgery were collected at day 1, week 1, months 1, 3, and 6, and yearly thereafter. Base-out prism glasses were recommended to patients with persistent diplopia associated with postoperative residual deviation < 10 PD at the latest visits after the operation. Reoperation was recommended if constant ocular deviation ≥ 10 PD persisted for six months after surgery and the patient had significant double vision or cosmetic concerns.

We carried out statistical analyses using the Statistical Analysis System version 9.4 (SAS Institute, Inc., Cary, NC, USA). We used the Wilcoxon rank sum test to compare the age at the onset and at surgery, the duration of the symptoms and diplopia, the angle of ocular deviation at distant and near fixation before and after MR recession surgery, and follow-up duration between the two groups. We used Chi-squared test to compare gender and Fisher’s exact test to compare use of prism glasses before surgery between the two groups. We used linear regression analysis to compare surgical dose–response between the two groups. A *p* <0.05 was considered statistically significant.

## Results

Fifteen DIE patients and 38 non-DIE patients were included in the study. The mean age at surgery was 38 ± 18 years (range, 15 to 65 years) in the DIE group, and 30 ± 15 years (range, 15 to 65 years) in the non-DIE group (*p* = 0.145). Other baseline features of both groups are displayed in Table 2. Eleven patients among the 15 DIE patients had myopia with a mean spherical equivalent refractive error of both eyes from -0.5 diopters to -5.5 diopters. Among the 38 non-DIE patients, 30 had myopia from -0.5 diopters to -6.8 diopters. There was no statistically significant difference in refraction between the two groups. Deviation at distant and near fixation was smaller in the DIE group than in the non-DIE group (*p* < 0.001). All patients in the DIE group had diplopia preoperatively, and 23 (61%) out of 38 patients had diplopia in the non-DIE group. The mean age of onset of diplopia was 34 ± 18 years (range, 14 to 63 years) in the DIE group and 30 ± 19 years (range, 7 to 64 years) in the non-DIE group (*p* = 0.317). The mean duration of diplopia was 46 ± 83 months (range, 6 to 340 months) in the DIE group and 24 ± 20 months in the non-DIE group (range, 6 to 96 months) (*p* = 0.570). Other patients had no diplopia. All patients with diplopia underwent neuroimaging, and no etiologic pathology was identified in their imaging.

**Table 2:** Baseline characteristics of patients with divergence insufficiency esotropia and non-accommodative esotropia without divergence insufficiency.

Variable	DIE (*n* = 15)	Non-DIE (*n* = 38)	*P*-value
Age at the surgery (years)	38 ± 18	30 ± 15	0.145^a^
Gender (male/female) (number)	8/7	21/17	1.000^b^
Duration of symptom (months)	46 ± 83	63 ± 74	0.189^a^
SE refractive errors (diopters)	-2.51 ± 2.18	-2.67 ± 2.48	0.832^a^
Use of prism glasses before strabismus surgery (number)	3 (20%)	3 (8%)	0.334^c^
Esodeviation (PD)			
at far distance fixation	25 ± 11	35 ± 10	<0.001^a^
at near distance fixation	9 ± 13	35 ± 11	<0.001^a^

*DIE* divergence insufficiency esotropia, *Non-DIE* non-accommodative esotropia without divergence insufficiency, *SE* spherical equivalent, *PD* prism diopters

^a^Wilcoxon rank sum test

^b^Chi-squared test

^c^Fisher’s exact test

Adjustment was carried out in 14 of the 15 DIE patients and 28 of the 38 non-DIE patients. Data on the details of surgery are presented in Table 3.

**Table 3:** Amount of surgery performed for divergence insufficiency esotropia and non-accommodative esotropia without divergence insufficiency (mm).

Preoperative deviation	DIE		Non-DIE	
	Total amount of surgery	(*n*)	Total amount of surgery	(*n*)
18 PD	14.0	(1)		
20–24 PD	14.0±0.3	(9)	9.3±2.8	(5)
25 PD	15.0±1.4	(2)	10.3±1.5	(6)
30 PD	15.0	(1)	10.5±0.8	(7)
35 PD			10.4±0.9	(7)
40 PD	13.5	(1)	11.7±0.4	(5)
45 PD			11.9±0.9	(4)
50 PD			13.0±0.7	(2)
60 PD	16	(1)	13.5±0.7	(2)

DIE, divergence insufficiency esotropia; Non-DIE, non-accommodative esotropia without divergence insufficiency; n, number of patients; PD, prism diopters

The mean follow-up duration was 8 ± 10 months (range, 1 to 40 months) in the DIE group and 17 ± 16 months (range, 1 to 64 months) in the non-DIE group (*p* = 0.036). In the patients with DIE, the mean preoperative horizontal angle of deviation in the primary position was 25 ± 11 PD compared with 3 ± 8 PD at the final postoperative visit. At the final visit, two patients had intermittent esotropia of 6 to 8 PD in the primary position with intermittent diplopia, and one patient who had esotropia of 60 PD preoperatively showed esophoria of 30 PD postoperatively, but didn’t complain of diplopia. The other 12 patients were orthophoric and didn’t have diplopia. There was no patient with postoperative overcorrection.

In the non-DIE group, the mean preoperative angle of deviation in the primary position was 35 ± 10 PD, compared with 3 ± 4 PD at the final postoperative visit. At the final visit, two patients had esotropia of 10 PD and 12 PD, and 14 patients had residual esodeviation of less than 10 PD. Among the non-DIE patients with residual esodeviation, four patients had postoperative intermittent diplopia. Among these four patients, none wanted to use prism glasses to correct their intermittent diplopia. The remaining patients with residual esodeviation did not have double vision. There was one patient with postoperative exodeviation of 2 PD.

The average surgical dose–response was 1.56 PD/mm in the DIE group and 2.91 PD/mm in the non-DIE group (*p* < 0.001).

In univariate analysis for surgical dose–response in the DIE group, greater preoperative ocular deviation at distant and near fixation (P = 0.003 and P = 0.03, respectively) was a significant factor associated with greater surgical dose–response. In the non-DIE group, greater preoperative ocular deviation at distant and near fixation (both *p* < 0.001) was also a significant factor associated with greater surgical dose–response.

## Discussion

This is one of just a few studies that describe the clinical characteristics of DIE patients and compare the surgical dose–response in DIE patients with that in non-DIE patients. The age range of DIE patients was 15 to 65 years. Distance esotropia associated with divergence insufficiency can occur at any age [13]. MRI studies have suggested that disruption of the LR-SR band may play a key pathogenic role in patients with distance esotropia and high myopia, irrespective of age [14]. In 1937, Pragen and Koch reviewed the clinical features of 54 patients with DIE [15]. The patients’ ages ranged from 20 to 45 years, and most of the patients had occupations that required extensive near work. In 2004, Webb et al. reported 26 cases with acquired distance esotropia associated with varying degrees of myopia from -0.75 to -10.0 diopters [16]. In our study, the majority of patients with DIE had myopia, although there was no statistically significant difference in the mean spherical equivalent refraction when compared with the non-DIE group. Because we did not routinely obtain systematic information about near work, we could not find out if intensive near work was associated with DIE, although most of the DIE patients recalled that they did prolonged close work before the symptoms of DIE developed. Other mechanisms, including progressive myopathy, could be related to the development of DIE [16]. Considering the varying degrees of myopia associated with DIE and the broad range of ages in these patients, many different mechanisms could underlie these phenomena.

With a relatively large surgical dosage, all DIE patients showed satisfactory postoperative results. Two patients had tolerable intermittent diplopia. The average surgical dose–response was 1.56 PD/mm in the DIE group and 2.91 PD/mm in the non-DIE group. The relatively low surgical dose-response to MR recession in the DIE group in this study generally agrees with findings in previous reports [8–11].

In univariate analysis for surgical dose–response in the DIE group, greater preoperative ocular deviation at distant or near fixation was a significant factor associated with greater surgical dose–response in both groups. The result was consistent with findings reported by Ridely-Lane et al.[9].

It may be important to differentiate between DIE and the term ‘age-related distance esotropia (ARDE)’ [2] because they exhibit similar clinical presentations. ARDE is known to occur in patients over 60 years old and may be related to orbital connective tissue degeneration because of aging [17]. Several characteristics, such as subacute onset and relatively young age at onset, were suggested as ways to differentiate DIE from ARDE in previous reports [18,19]. However, it is still difficult to clearly differentiate these pathologies. There were two patients older than 60 in the DIE group in this study. Both of them had acute onset diplopia. Cranial and orbital MRI results were normal in both eyes. However, the images were not targeted to detect connective tissue degeneration in the orbit. Either patient in the DIE group in this study might have had ARDE or mixed components of DIE and ARDE. We hope that definite diagnostic criteria are established for these disease categories in the near future.

There were several limitations in this study. First, the DIE group was small because of the relatively rare occurrence of DIE. In addition, the follow-up duration after surgery was relatively short in this study. Further study with a larger sample and longer-term follow-up duration is needed to confirm the results of this study. Second, the adjustment procedure was more frequently done during the operation in the DIE group than in the non-DIE group, which might have decreased the final surgical amount in the non-DIE group. Third, baseline ocular deviation was smaller in the DIE group than in the non-DIE group, which might have caused underestimation of the difference in surgical dose-response between the two groups, because strabismus surgery tends to produce more change in alignment when the deviation is large and less when it is small [20]. Fourth, although relatively uniform manipulations for surgery were followed in our hospital, the fact that there were two different surgeons might have introduced some differences in the data. Fifth, the surgery was not based on the dynamic angle of deviation. Fifth, this study was retrospective; so, although the postoperative follow-up schedule was relatively uniform, there were some differences in follow-up intervals between a few patients. A prospective study with uniform protocols is needed to confirm the actual difference in surgical response between the two diseases. Sixth, our study population was recruited from a single institution, and all the participants were of the same race. Therefore, the results might not be valid in patients of a different ethnicity.

In conclusion, this study revealed that surgical dose–response was significantly lower in the DIE patients than in the non-DIE patients. Thus, augmented MR recession surgery could be regarded as an effective treatment option for patients with DIE. Further study with a larger sample and long-term follow-up is needed to confirm the results of this study and seek the proper extent of surgery in these patients.
